# Hemophilia A and B mice, but not VWF^−/−^mice, display bone defects in congenital development and remodeling after injury

**DOI:** 10.1038/s41598-019-50787-9

**Published:** 2019-10-08

**Authors:** Sarah Taves, Junjiang Sun, Eric W. Livingston, Xin Chen, Jerome Amiaud, Regis Brion, William B. Hannah, Ted A. Bateman, Dominique Heymann, Paul E. Monahan

**Affiliations:** 1 Department of Biomedical Engineering, University of North Carolina, Chapel Hill, NC USA; 2 Global Research, Novo Nordisk A/S, Maløv, Denmark; 3 Gene Therapy Center, University of North Carolina, Chapel Hill, NC USA; 4 Division of Molecular Pharmaceutics, Eshelman School of Pharmacy, University of North Carolina, Chapel Hill, NC USA; 5 INSERM, U1238, Faculty of Medicine, Université de Nantes, Nantes, F-44093 France; 6 Department of Radiation Oncology, University of North Carolina, Chapel Hill, NC USA; 7 INSERM, U1232, CRCiNA, Institut de Cancérologie de l’Ouest, Université de Nantes, Université d’Angers, Saint-Herblain, F-44805 France; 8 University of Sheffield, INSERM, Associated European Laboratory Sarcoma Research Unit, Department of Oncology and Metabolism, Sheffield, S10 2RX UK; 9 Harold R. Roberts Comprehensive Hemophilia Diagnosis and Treatment Center, University of North Carolina, Chapel Hill, NC USA; 10 Spark Therapeutics, Philadelphia, PA USA

**Keywords:** Bone, Haematological diseases

## Abstract

While joint damage is the primary co-morbidity of hemophilia, osteoporosis and osteopenia are also observed. Coagulation factor VIII deficient (FVIII^−/−^) mice develop an osteoporotic phenotype in the absence of induced hemarthrosis that is exacerbated two weeks after an induced joint injury. Here we have compared comprehensively the bone health of clotting factor VIII, factor IX, and Von Willebrand Factor knockout (FVIII^−/−^, FIX^−/−^, and VWF^−/−^ respectively) mice both in the absence of joint hemorrhage and following induced joint injury. We found FVIII^−/−^ and FIX^−/−^ mice, but not VWF^−/−^ mice, developmentally have an osteoporotic phenotype. Unilateral induced hemarthrosis causes further bone damage in both FVIII^−/−^ and FIX^−/−^ mice, but has little effect on VWF^−/−^ bone health, indicating that the FVIII.VWF complex is not required for normal bone remodeling *in vivo*. To further investigate the bone healing following hemarthrosis in hemophilia we examined a two week time course using microCT, serum chemistry, and histological analysis. Elevated ratio of osteoprotegerin (OPG)/receptor activator of nuclear factor-kappa B ligand (RANKL), increased osterix^+^ osteoblastic cells, and decreased smoothness of the cortical bone surface were evident within several days of injury, indicative of acute heterotopic mineralization along the cortical surface. This was closely followed by increased interleukin-6 (IL-6) levels, increased osteoclast numbers, and significant trabecular bone loss. Uncoupled and disorganized bone formation and resorption continued for the duration of the study resulting in significant deterioration of the joint. Further elucidation of the shared mechanisms underlying abnormal bone homeostasis in the absence of FVIII or FIX is needed to guide evidence-based approaches to the screening and treatment of the prevalent bone defects in hemophilia A and B.

## Introduction

Hemophilia A and hemophilia B are X-linked recessive inherited bleeding disorders caused by deficient activity of blood coagulation factors VIII (FVIII) and IX (FIX), respectively, resulting in severely deficient thrombin generation. Severe hemophilia is associated with chronic degenerative joint and bone disease^1–3^. Although the primary crippling, painful symptoms are experienced at the joint articulation, osteopenia and osteoporosis in individuals with hemophilia also are widely documented^4–8^. Recent studies of hemophilia A mice suggest that increased bone resorption and low bone mineral density may be associated with factor VIII deficiency even in the absence of gross hemarthrosis^9,10^.

Tight control of bone structure and phosphocalcic balance is essential to maintain skeletal integrity and depends upon the proper coordination of bone osteoclast and osteoblast activity. Bone remodeling, particularly following injury, can be rapidly modulated by a number of factors including pro-inflammatory mediators present due to injury and the final molecular effectors receptor activator of nuclear factor-kappa B (RANK), RANK ligand (RANKL), and osteoprotegerin (OPG), the natural decoy receptor for RANKL^11,12^.

Von Willebrand factor (VWF) is a multimeric protein that is required for platelet adhesion and whose lack results in von Willebrand Disease (VWD), the most common coagulation disorder in humans. VWF also serves an important function as a carrier protein for FVIII in circulation, decreasing the clearance of FVIII from plasma. Recent work has demonstrated that the FVIII.VWF complex associates physically with OPG^13,14^ and RANKL, and inhibits osteoclastogenesis via interaction with these mediators^15^. The FVIII.VWF complex may act as an anti-resorption factor both in normal physiology and following hemarthrosis. An increased incidence of low bone mineral density, osteoporosis, or abnormal structural integrity of bone has not been described in humans with VWD.

Utilizing mouse FVIII, FIX, and VWF gene knockout disease models (FVIII^−/−^ FIX^−/−^ VWF^−/−^, respectively, and their WT littermate controls), we compared congenital bone structure as well as remodeling phenotypes following joint hemorrhage. The scope of our studies sought to model the observed clinical association of severe deficient factor VIII and bone abnormalities in males with hemophilia A. Using dual energy X-ray absorptiometry (DXA) and microcomputed tomography (microCT) measurement we found that in the absence of any observed injury or bleeding FVIII^−/−^ mice display significantly low bone mineral density (BMD) and other deficits of trabecular bone integrity. FVIII is a cofactor for the protease factor IXa in the complex that activates factor X, leading to the ultimate amplification of thrombin generation. Therefore we also hoped to dissect whether the absence of the FVIII protein itself, or absence of an intact complex of VWF protein with FVIII protein, were necessary to promote the abnormal bone phenotype previously described in male hemophilia mice and in males clinically. We examined in parallel the phenotype that develops in age-matched male mice that express FVIII but have isolated complete absence of FIX or have isolated complete absence of VWF. Our findings demonstrate that mice congenitally deficient for either factor VIII or factor IX demonstrate similar deficits of BMD and trabecular bone integrity. Further, despite the close physiologic association of VWF with FVIII, we report that male VWF^−/−^ mice do not display this congenital abnormal bone phenotype.

Hemarthrosis exacerbated these congenital BMD and structural differences in hemophilia mice. At two weeks post-injury, FVIII^−/−^ and FIX^−/−^ mice exhibited drastic trabecular bone loss and large areas of heterotopic mineralization of the cortical bone surface. These observations prompted a time course study of early bone response to joint hemorrhage in FVIII^−/−^ mice. This examination demonstrated acute heterotopic bone formation within days of injury, closely followed by bone resorption beginning one week following hemarthrosis. VWF^−/−^ animals did not display the same characteristics, leading to the conclusion that an intact FVIII/VWF complex is not required for bone remodeling post-injury.

## Results

**Figure 1:**
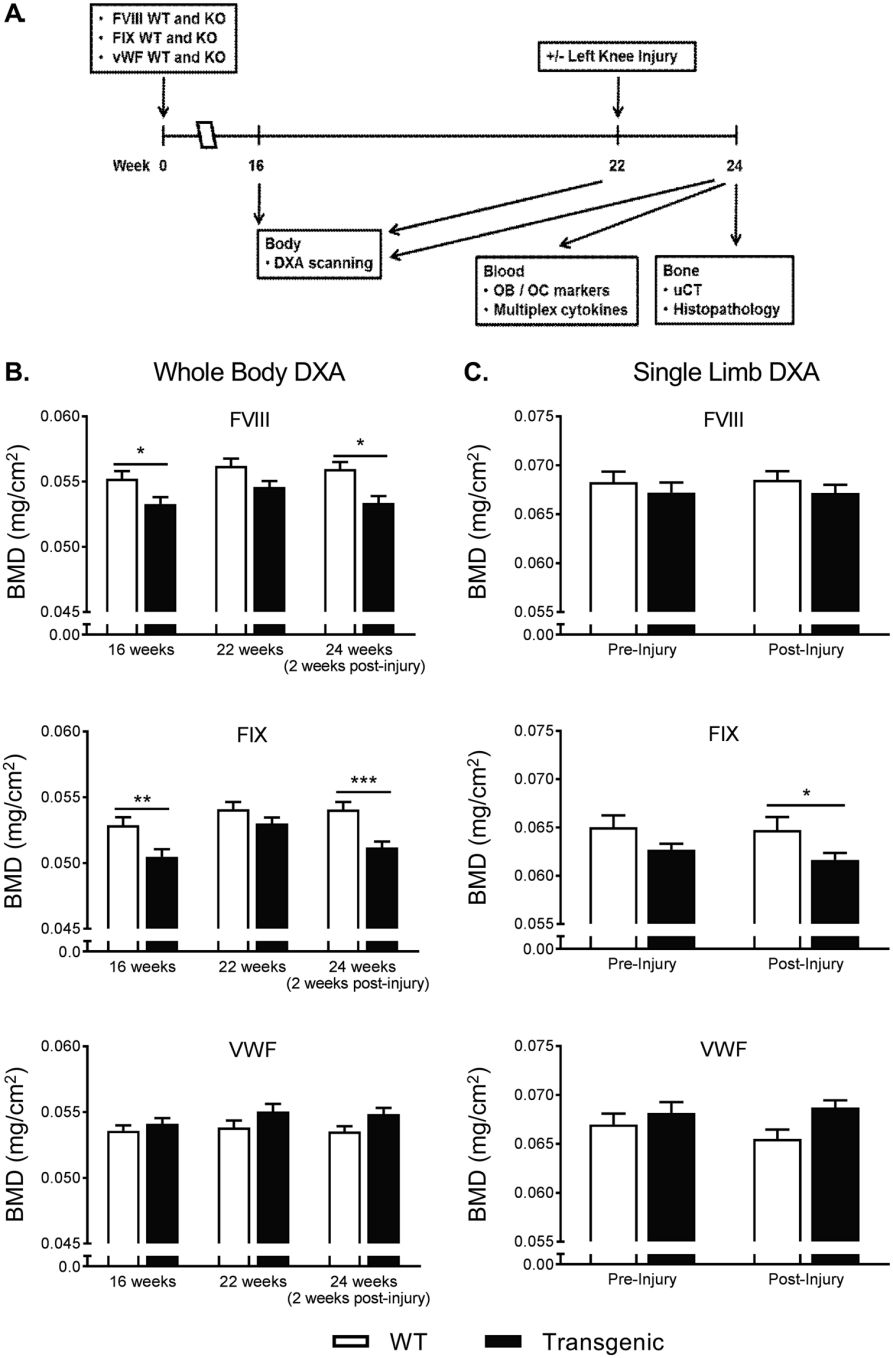
Experimental design and DXA quantification of BMD in FVIII^−/−^ and FIX^−/−^ mice and VWF^−/−^ mice and their respective wild type (hemostatically normal) littermates. (**A**) Diagram of the experimental design. (**B**) Whole body DXA measurements were taken at 16, 22, and 24 weeks in FVIII (FVIII^−/−^ n = 34; WT n = 26), FIX (FIX^−/−^ n = 29; WT n = 26), and VWF (VWF^−/−^ n = 27; WT n = 19) lines of mice. FVIII^−/−^ and FIX^−/−^ mice showed significantly decreased BMD at 16 weeks with some recovery by 22 weeks of age. Two weeks following joint-injury whole body BMD dropped significantly in FVIII^−/−^ and FIX^−/−^ mice. VWF^−/−^ mice showed no difference from WT littermates at any time point, nor following injury. (**C**) Single limb DXA measurements were unable to detect significant BMD changes when comparing pre- to post-injury BMD in any line of mice, knockout or WT (FVIII^−/−^ n = 34/WT n = 27; FIX^−/−^ n = 19/WT n = 29; VWF^−/−^ n = 28/WT n = 18); However, FIX^−/−^ animals displayed significantly lower BMD of the injured limb compared to their WT littermate controls after injury. A non-parametric two-way analysis of variance (Kruskal–Wallis) was performed followed by a Sidak post-hoc test. Average ± SEM. *P < 0.05, **P < 0.01, ***P < 0.001.

To compare the bone remodeling phenotypes of FVIII^−/−^, FIX^−/−^, and VWF^−/−^ mice we performed a single left knee injury at the time of skeletal maturity. BMD was assessed by DXA twice prior to injury at 16 and 22 weeks to examine normal bone growth, and at two weeks post-injury, 24 weeks old, to assess bone damage following hemarthrosis. Histopathology, µCT evaluation, and a cytokine multiplex assay were performed two weeks post-injury (Fig. 1A).

### FVIII^−/−^ and FIX^−/−^, but not VWF^−/−^ mice, display a congenital phenotype of significantly reduced whole body BMD and abnormal bone structural features, compared to WT littermates

Between 16 and 22 weeks of age all mice show absolute increases in whole body BMD likely corresponding to normal growth. Prior to injury, both FVIII^−/−^ and FIX^−/−^ mice have low BMD and low BMC at 16 weeks, compared to their WT littermates, as measured by whole body DXA. The difference in BMD at 16 weeks is 3.44% lower in FVIII^−/−^ mice than WT littermates (*p* = 0.033). The difference in BMD at 16 weeks is 4.54% lower in FIX^−/−^ mice than WT littermates (*p* = 0.0057). When examined at 22 weeks of age, mean BMD and BMC were lower in FVIII and FIX deficient mice, however the differences from WT littermates were statistically different only for BMC in the FIX^−/−^ mice. Unlike FVIII^−/−^ and FIX^−/−^ mice, VWF^−/−^ mice showed no difference in whole body BMD compared to their WT littermates at any time point (Table 1, Fig. 1B).

**Table 1:** Comparison of congenital differences in FVIII, FIX, and VWF lines of mice.

	FVIII^−/−^			FIX^−/−^			VWF^−/−^		
	WT	KO	Significance	WT	KO	Significance	WT	KO	Significance
**Trabecular μCT Parameters**									
vBMD (mgHA/cm^3^)	174.9096 +/− 12.149	126.4247 +/− 10.333	0.0057	181.8782 +/− 10.565	153.7318 + /− 8.157	0.0447	172.7436 +/− 11.854	179.0456 +/− 10.382	0.7028
Trabecular Bone Volume Fraction BV/TV (%)	0.1445 +/− 0.011	0.1038 +/− 0.0009	0.0080	0.1468 +/− 0.010	0.1227 +/− 0.008	0.0654	0.1378 +/− 0.011	0.1404 +/− 0.011	0.7445
Connectivity Density (mm^3^)	81.9748 +/− 9.970	54.565 +/− 7.649	0.0418	114.9248 +/− 10.401	84.3252 +/− 7.693	0.0258	111.0571 +/− 8.879	120.4066 +/− 10.003	0.5323
Trabecular Thickness (mm)	0.0530 +/− 0.0002	0.0503 +/− 0.002	0.2728	0.0454 +/− 0.001	0.0457 +/− 0.002	0.5795	0.0436 +/− 0.0001	0.0447 +/− 0.001	0.5537
Trabecular Number (mm^-1^)	4.2003 +/− 0.146	3.5781 +/− 0.165	0.0084	4.916 +/− 0.127	4.2954 +/− 0.177	0.0082	4.7223 +/− 0.121	4.9193 +/− 0.095	0.2166
Trabecular Spacing (mm)	0.2352 +/− 0.010	0.2843 +/− 0.016	0.0110	0.1963 +/− 0.006	0.2328 +/− 0.011	0.0081	0.2050 +/− 0.006	0.1948 +/− 0.005	0.2039
**Cortical mCT Parameters**									
Cortical Thickness	0.2175 +/− 0.003	0.2197 +/− 0.004	0.6642	0.2019 +/− 0.003	0.1960 +/− 0.003	0.1535	0.1940 +/− 0.005	0.1925 +/− 0.003	0.7643
Cortical Porosity	3.6481 +/− 0.107	3.6946 +/− 0.044	0.2717	4.1843 +/− 0.073	4.4157 +/− 0.218	0.3236	2.914 +/− 0.567	2.400 +/− 0.411	0.4628
Marrow Cavity Area	0.9762 +/− 0.0309	0.9014 +/− 0.32	0.1036	1.0182 +/− 0.048	1.0330 +/− 0.054	0.8384	1.2221 +/− 0.031	1.1087 +/− 0.031	0.0217
Polar Moment of Inertia	0.4581 +/− 0.020	0.4152 +/− 0.018	0.1215	0.4328 +/− 0.027	0.4137 +/− 0.026	0.6149	0.5228 +/− 0.20	0.4614 +/− 0.017	0.0293
Surface of Articulating Bone Ratio of Smoothness	0.8989 +/− 0.003	0.8942 +/− 0.007	0.5404	0.9049 +/− 0.004	0.9099 +/− 0.003	0.3529	0.9179 +/− 0.002	0.9127 +/− 0.002	0.1635
**DXA Parameter**s									
BMD 16 weeks	0.0552 +/− 0.001	0.0533 +/− 0.001	0.0325	0.0529 +/− 0.001	0.0505 +/− 0.001	0.0057	0.0536 +/− 0.001	0.0541 +/− 0.001	0.8318
BMD 22 weeks	0.0562 +/− 0.001	0.0546 +/− 0.001	0.1010	0.0541 +/− 0.001	0.0530 +/− 0.001	0.3946	0.0538 +/− 0.001	0.0551 +/− 0.001	0.2229
BMC 16 weeks	0.4692 +/− 0.009	0.4412 +/− 0.007	0.0500	0.4106 +/− 0.100	0.3778 +/− 0.007	0.0113	0.4477 +/− 0.008	0.4558 +/− 0.007	0.8784
BMC22 weeks	0.4889 +/− 0.009	0.4674 +/− 0.007	0.1566	0.4306 +/− 0.009	0.3962 +/− 0.006	0.0068	0.4625 +/− 0.008	0.4674 +/− 0.010	0.9685

µCT measurements were compared using *t*-test comparison between WT and KO of each line. FVIII WT n = 16, KO n = 14; FIX WT n = 14, KO n = 14; VWF WT n = 10, KO n = 17. DXA measurements BMD and BMC were compared using 2-way ANOVA with multiple comparisons. FVIII WT n = 26, KO n = 34; FIX.

Confirmatory examination was performed using microCT of the proximal tibia. FVIII^−/−^ deficient mice at skeletal maturity (22–24 weeks of age) had a 27.8% lower measured vBMD at baseline than WT littermates (*p* = 0.0057). FIX^−/−^ mice had 15.5% lower vBMD at baseline than WT littermates, in the absence of injury or observed hemorrhage (*p* = 0.0447). VWF^−/−^ mice did not differ in vBMD from WT littermates, as measured by microCT. MicroCT examination of additional measures of trabecular integrity also demonstrated that significant parallel deficits exist in FVIII^−/−^ and FIX^−/−^ mice. FVIII^−/−^ mice displayed a 17.3% increase in the trabecular space, associated with a 33.4% decrease in trabecular bone connectivity density and a 14.8% decrease in trabecular number. Uninjured FIX^−/−^ mice had similar differences of 18.6%, 26.6%, and 12.7% in these respective parameters, when compared to uninjured WT littermates. VWF^−/−^ mice at skeletal maturity did not differ in measures of trabecular bone health from WT littermates (Table 1).

### Two weeks following induced joint injury, injured limbs of FVIII^−/−^, FIX^−/−^ exhibit elevated synovitis scores compared to the non-injured limb and to injured WT littermate controls

**Figure 2:**
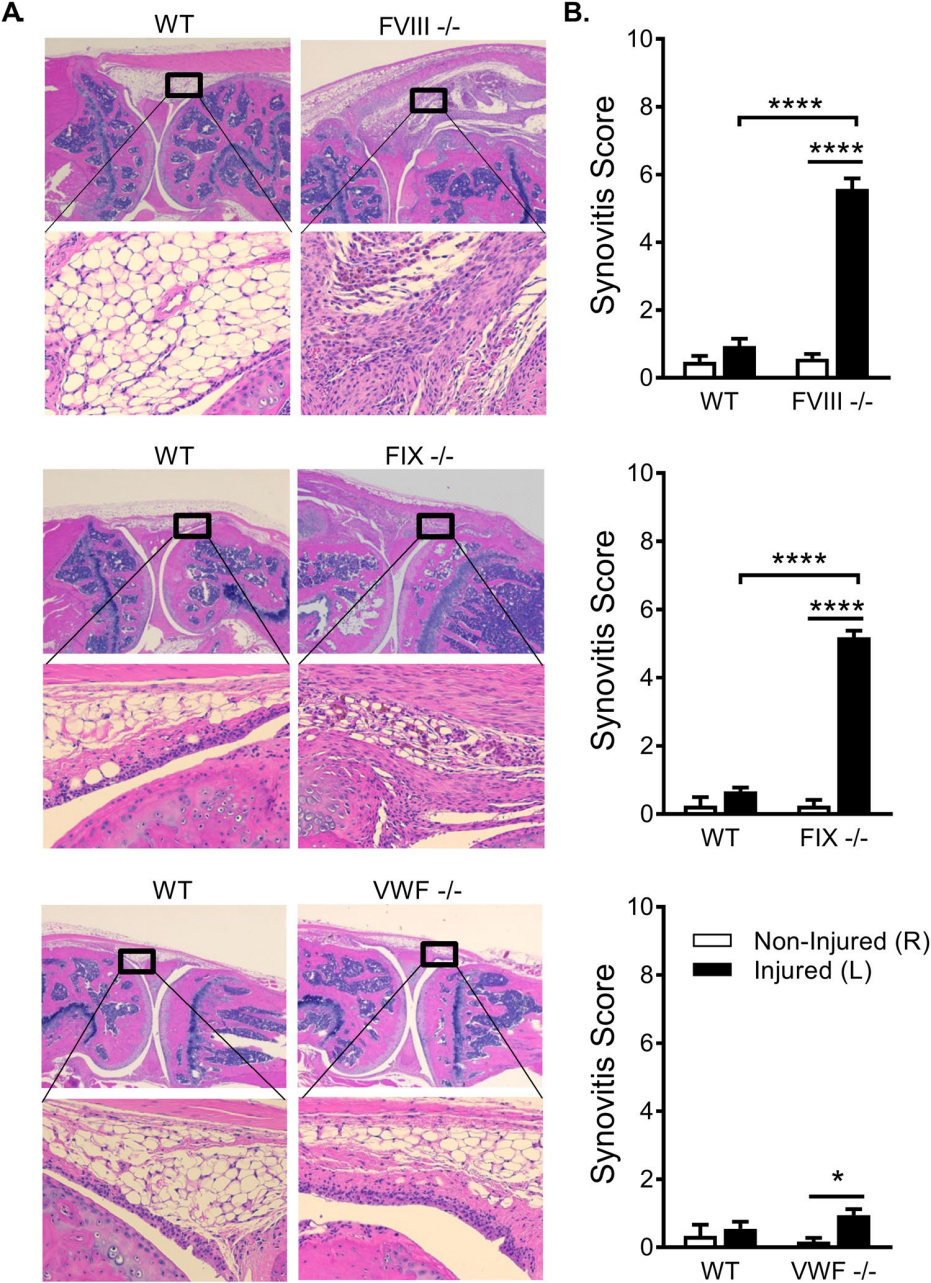
Knee injury produced increased soft tissue damage in the joints of FVIII^−/−^ and FIX^−/−^, but not VWF^−/−^ mice. (**A**) Representative images of joint histology two weeks following induced hemarthrosis. Imaging was performed on a Nikon Microphor SA microscope equipped with 10/0.30, 20/0.50, and 40/0.70 numeric aperture objective lenses. Images were captured with a DMX-1200 color camera using the Act-1 software (entire system from Nikon Instruments, Melville, NY). Representative images are shown at original magnification ×40 (top) and ×200 (bottom). (**B**) Soft tissue damage was assessed for the non-injured (right) limb and injured (left) limb two weeks post-injury in FVIII, FIX, and VWF lines of mice. Histological sections were scored using a murine hemophilic synovitis grading system consisting of a 0-to-10 point scale that quantitates evidence of hyperplasia of the synovial lining cells and of increased synovial vasculature replacing the subsynovial space, as well as the presence of blood, hemosiderin, cartilage erosion, or synovial villous formation. The injured limbs of FVIII^−/−^ and FIX^−/−^ mice showed significantly elevated scores at two weeks post-injury compared to the non-injured limb and to injured WT littermate controls. The injured limbs of VWF^−/−^ mice were not significantly different from injured WT or non-injured controls. Average scores with SEM are shown. *p < 0.05; ****p < 0.001. FWIII^−/−^ groups (Non-injured: WT n = 7/FVIII^−/−^ n = 10; Injured: WT n = 22/FVIII^−/−^ n = 23); FIX^−/−^ groups (Non-injured: WT n = 4/FIX^−/−^ n = 8; Injured: WT n = 16/FIX^−/−^ n = 9); VWF^−/−^ groups (Non-injured: WT n = 3/VWF^−/−^ n = 12; Injured: WT n = 12/VWF^−/−^ n = 20).

The histopathological features of hemophilic synovitis following knee joint bleeding challenge were quantified by the Valentino scale^16^, a validated murine hemophilic synovitis grading system that quantitates increasing pathology on a 0-to-10 point scale. All non-injured, hemostatically normal mice scored between 1 and 0 on the modified Valentino scale. Following unilateral puncture of the knee joint capsule to induce haemorrhage, the same examiner measured the joint diameter serially over two weeks post-injury using a micro-caliper (Fig. 2). WT hemophilia mice and WT littermates of the mutant mice demonstrated minimal (mean < 5%, median = 0%) increase in joint diameter at 4 hours and 1 day after injury, with all WT mice returned to baseline thereafter. In contrast, from day 1 through day 7 after injury, most of the VWF^−/−^ mice demonstrated 5–15% increase in joint diameter over baseline. The greater extent of joint swelling and the more prolonged joint deformation are consistent with the development of bleeding in VWF^−/−^ mice and sub-acute persistence of joint involvement after induced bleeding. The joint swelling that developed in FVIII^−/−^ mice was qualitatively different from WT and similar to VWF^−/−^ in regards to the persistence of swelling throughout the first week following the induced hemorrhage. The mean % increase in diameter of the joint in FVIII^−/−^ mice at each time point days 1, 2, 3, 7 was 1.8 to 2.9 times greater than in the VWF^−/−^ mice. Swelling of the joint was still present in FVIII^−/−^ mice at two weeks after injury, at a time when joint diameter of VWF^−/−^ mice returned to normal. At two weeks post-injury, hemostatically normal littermate controls of the WT FVIII, FIX, and VWF deficient mice had mean scores of 0.94, 0.67, and 0.5 respectively, which is consistent with previous reports^17,18^, and demonstrates that joint histopathology had returned to normal following injury. The non-injured knockout groups, FVIII^−/−^, FIX^−/−^, and VWF^−/−^ also displayed little joint pathology and mean scores of 0.57, 0.25, and 0.17 respectively (Fig. 2A,B). In contrast, the mean scores of injured FVIII^−/−^ and FIX^−/−^ groups remained highly elevated two weeks post-injury at 5.57 and 5.18 respectively (p < 0.0001 in comparison to both WT injured and knockout non-injured, in both FVIII^−/−^ and FIX^−/−^ lines) (Fig. 2B). Injured VWF^−/−^ mice mean score was 0.95. While this was significantly elevated in comparison to non-injured VWF^−/−^ mice (p = 0.02), it was not significantly elevated compared to normal littermate controls of the WT VWF deficient mice that were either injured or non-injured animals and still falls in the 1 to 0 range considered normal.

### MicroCT scans revealed significant changes in surface morphometry and trabecular structure of the injured limb within the FVIII^−/−^ and FIX^−/−^ cohorts when compared to the contralateral control or WT littermates but not in VWF^−/−^ mice

**Figure 3:**
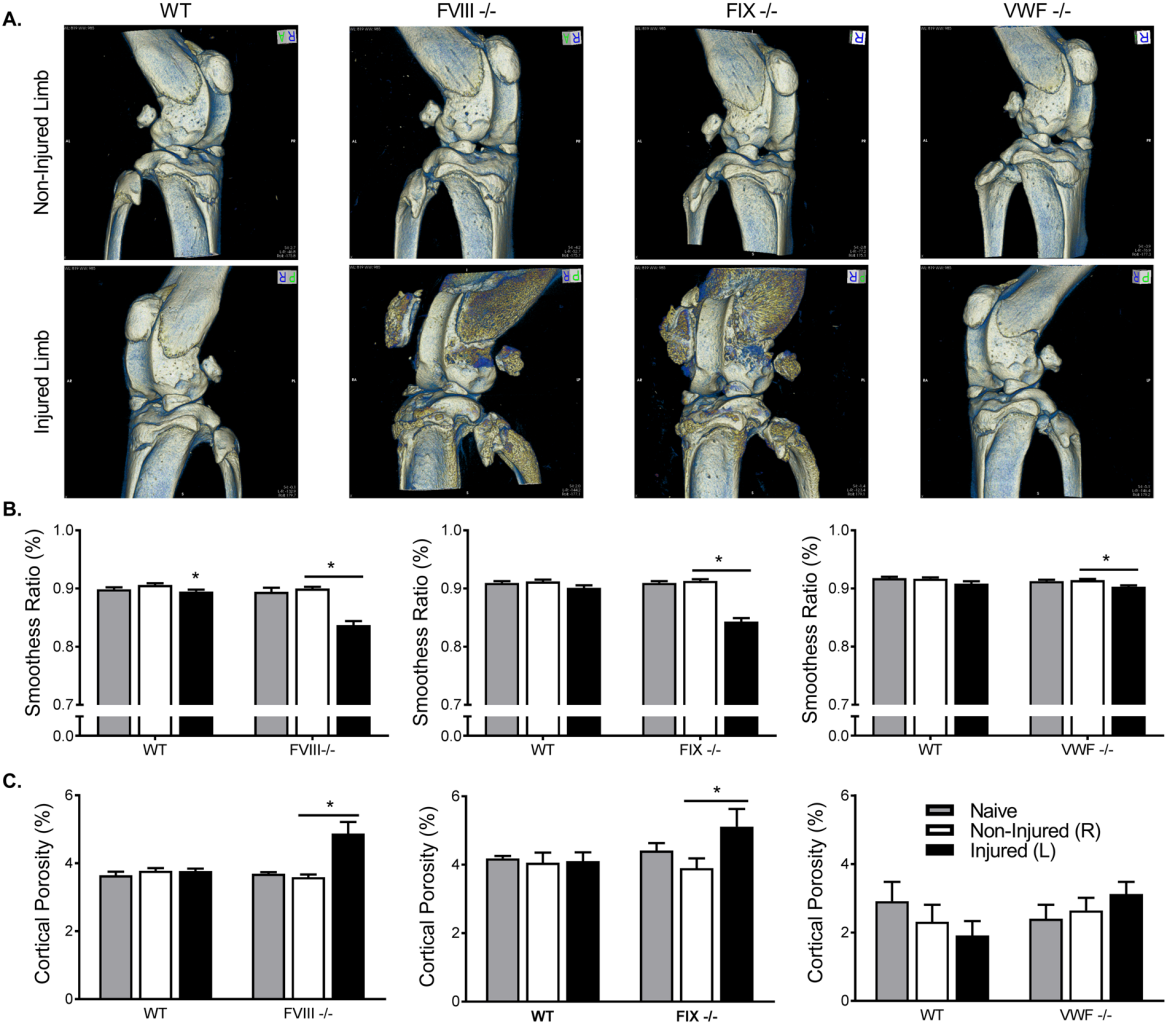
Hemarthrosis caused increased cortical porosity and surface roughness at the bone articulation in FVIII^−/−^ and FIX^−/−^, but not VWF^−/−^ mice. (**A**) Representative microCT images of cortical bone structure. (**B**) Smoothness ratio showed significant decreases following injury, particularly in FVIII^−/−^ (Naïve: WT n = 16/FVIII^−/−^ n = 18; non-injured: WT n = 18/FVIII^−/−^ n = 23; Injured: WT n = 18/FVIII^−/−^ n = 23) and FIX^−/−^ (Naïve: WT n = 14/FIX^−/−^ n = 14; non-injured: WT n = 17/FIX^−/−^ n = 18; Injured: WT n = 17/FVIX^−/−^ n = 18) mice. (**C**) Cortical porosity was significantly elevated following injury in FVIII^−/−^ and FIX^−/−^ mice, but not VWF^−/−^ (Naïve: WT n = 10/VWF^−/−^ n = 17; non-injured: WT n = 15/VWF^−/−^ n = 18; Injured: WT n = 15/VWF^−/−^ n = 17) mice. Average ± SEM. *p < 0.05.

Volumetric renderings show substantial mineral formation was noted at or near the articular surfaces of the joint, including those of the patella (Fig. 3A). The effects on all lines of WT mice and VWF^−/−^ transgenic mice appeared minimal. Surface smoothness analysis supported the visual findings, as the smoothness ratio was significantly reduced in the FVIII^−/−^ and FIX^−/−^ groups (−7% and −8%, respectively; Fig. 3B). Additionally, the increase in surface mineralization affected the structure of the cortical bone at the mid-diaphysis, causing substantial increases in porosity (+39% and +28%; Fig. 3C) for FVIII^−/−^ and FIX^−/−^ mice, respectively (Fig. 1, Supplemental data). There were no significant changes in porosity in the WT and VWF^−/−^ cohorts (Fig. 3C).

**Figure 4:**
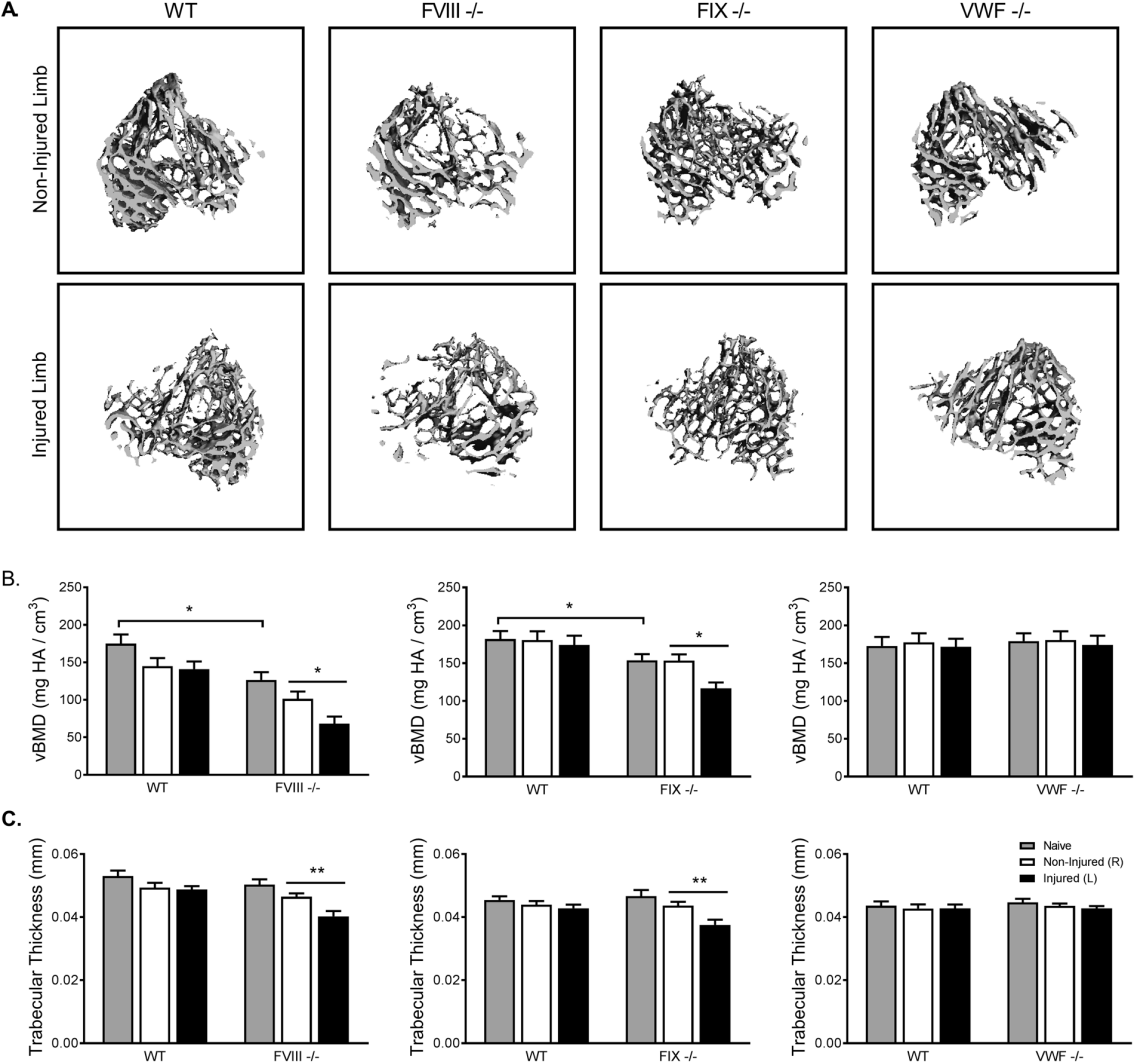
FVIII^−/−^ and FIX^−/−^ mice, but not VWF^−/−^ mice, had reduced trabecular bone in the absence of injury, which was further diminished in the injured limb following hemarthrosis. (**A**) Representative microCT images of trabecular structure. (**B**) Compared to WT littermate controls, FVIII^−/−^ and FIX^−/−^ mice had significantly reduced vBMD in the absence of injury. Joint injury resulted in further loss of vBMD in the injured limb two weeks post-injury. (**C**) Trabecular thickness was likewise reduced in the injured limb of FVIII^−/−^ and FIX^−/−^ mice. VWF^−/−^ mice displayed no differences from WT littermate trabecular bone health prior to or following injury. Average ± SEM. *p < 0.05; **p < 0.01. FVIII^−/−^ groups (Naïve: WT n = 15/FVIII^−/−^ n = 13; non-injured: WT n = 18/FVIII^−/−^ n = 25; Injured: WT n = 17/FVIII^−/−^ n = 25), FIX^−/−^ groups (Naïve: WT n = 19/FIX^−/−^ n = 18; non-injured: WT n = 19/FIX^/-^ n = 18; Injured: WT n = 19/FIX^−/−^ n = 18); VWF^−/−^ groups (Naïve: WT n = 10/VWF^−/−^ n = 17; non-injured: WT n = 15/VWF^−/−^ n = 24; Injured: WT n = 15/VWF^−/−^ n = 24).

Further microCT analyses revealed changes in both volume and structure of the trabecular bone in the proximal tibia following knee joint hemorrhage. Volumetric renderings of trabecular bone are shown in Fig. 4A. Within the FVIII^−/−^ cohort, the uninjured knockout animals showed a 28% reduction in vBMD relative to their WT littermates (Fig. 4B). A phenotypic difference was also present in the FIX^−/−^ animals (−15%), but not in the VWF^−/−^ group. The injured limbs from the FVIII^−/−^ and FIX^−/−^ mice each exhibited further deterioration in vBMD versus the contralateral control limbs (−30% and −24%, respectively), while the WT and VWF^−/−^ groups showed no differences. Though there were no phenotypic differences found in trabecular bone thickness, both FVIII^−/−^ and FIX^−/−^ cohorts showed significant differences in the injured limb versus the contralateral uninjured control limb (−13% and −14%, respectively; Fig. 4C). The injured limbs from the WT and VWF^−/−^ animals were again unaffected. As there were no findings that suggested an abnormal bone phenotype in the absence of VWF, no further investigation of VWF^−/−^ mice was performed.

### Injury produces significantly higher OPG/RANKL ratios in FVIII^−/−^ and FIX^−/−^ mice compared to WT littermate controls

**Figure 5:**
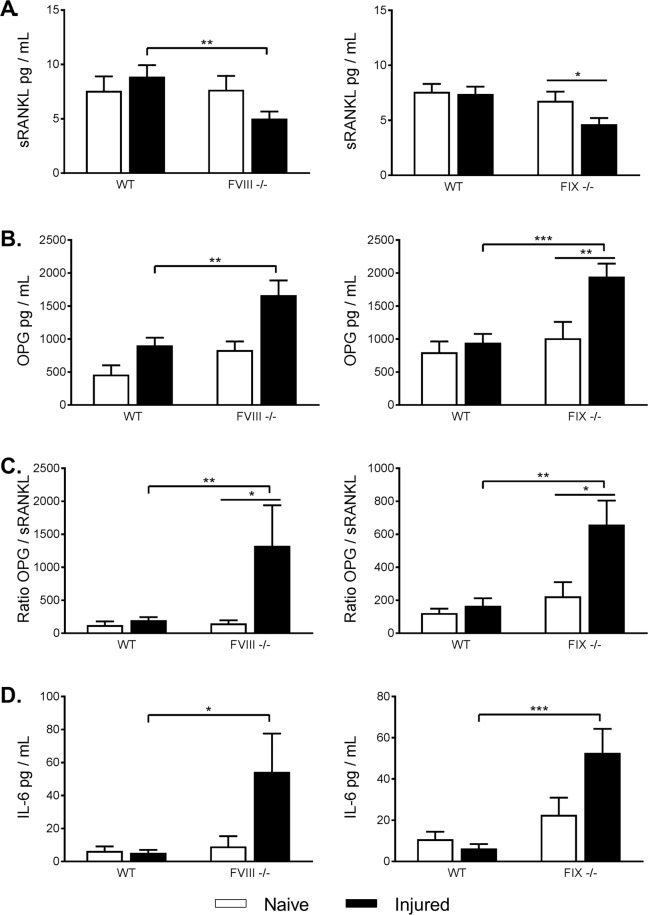
Following injury, OPG/sRANKL ratios and IL-6 were significantly elevated in FVIII^−/−^ and FIX^−/−^ mice compared to injured WT littermate controls. (**A**) sRANKL was significantly lower in injured FVIII^−/−^ and FIX^−/−^ mice in comparison to injured WT controls and naïve knockout mice, respectively. (**B**) OPG was significantly elevated in injured FVIII^−/−^ and FIX^−/−^ mice in comparison to injured WT mice, and in injured vs naïve FIX^−/−^ mice. (**C**) OPG/sRANKL ratios were significantly higher in injured FVIII^−/−^ and FIX^−/−^ mice in comparison to both naïve knockout and WT littermate controls. (**D**) IL-6 levels were significantly higher than injured WT controls. Average ± SEM. *p < 0.05; **p < 0.01; ***p < 0.001. FVIII^−/−^ groups (Non-injured: Naive n = 14/FVIII^−/−^ n = 27; Injured: Naive n = 14/FVIII^−/−^ n = 33); FIX^−/−^ groups (Non-injured: Naive n = 13/FIX^−/−^ n = 13; Injured: Naive n = 18/FIX^−/−^ n = 27). Number of outliers removed: FVIII^−/−^ groups (Non-Injured: Naive n = 1; Injured: Naïve n = 3; FVIII^−/−^ n = 2); FIX^−/−^ groups (Injured: Naive n = 5/FIX^−/−^ n = 4).

To investigate the acute heterotopic mineralization at the cortical bone surface and loss of trabecular bone in FVIII^−/−^ and FIX^−/−^ mice, we examined the serum levels of the final effector of bone resorption, RANKL and OPG, its decoy receptor. Two weeks following knee joint hemorrhage injured FVIII^−/−^ mice showed significantly lower levels of RANKL and increased expression of OPG compared to uninjured FVIII^−/−^ controls. Injured WT littermates also showed a significant increase in OPG but only a mild increase in RANKL expression compared to naïve controls. This leads to a 7.4 fold higher OPG/RANKL ratio in injured FVIII^−/−^ compared to injured WT or naïve controls (p = 0.0026) (Fig. 5A–C). Similarly, injured FIX^−/−^ mice showed a significantly lower RANKL and significant higher OPG expression compared naïve FIX^−/−^ controls, while WT littermates showed no significant effect of injury on RANKL or OPG expression, resulting in a 4-fold higher OPG/RANKL ratio in injured FIX^−/−^ mice (p = 0.0004) (Fig. 5A–C). The high OPG/RANKL ratios in FVIII^−/−^ and FIX^−/−^ mice mark a significant swing towards the inhibition of osteoclastogenesis and a shift away from bone resorption at two weeks following injury.

The pro-inflammatory cytokine IL-6 is known to have potent effects on bone remodeling both independently and through production of both RANKL and OPG. While IL-6 can also act directly on osteoblasts to promote differentiation^19^, it’s most potent direct action is on osteoclasts as a pro-resorption factor^20^. At two weeks post-injury we found significant IL-6 elevations in both the FVIII^−/−^ and FIX^−/−^ injury groups in comparison to injured WT littermates, 6.2 and 8.3 fold higher respectively (FVIII^−/−^ p = 0.0441; FIX^−/−^ p = 0.0006) (Fig. 5D). Here we focused on cytokine measurements similar to FVIII^−/−^ and FIX^−/−^ mice because these are related to the histological and bone endpoints examined in this manuscript. Additional cytokine measurements are reported in Supplemental Tables 1 and 2.

### Bone formation along the cortical bone surface is closely followed by bone resorption after hemarthrosis

**Figure 6:**
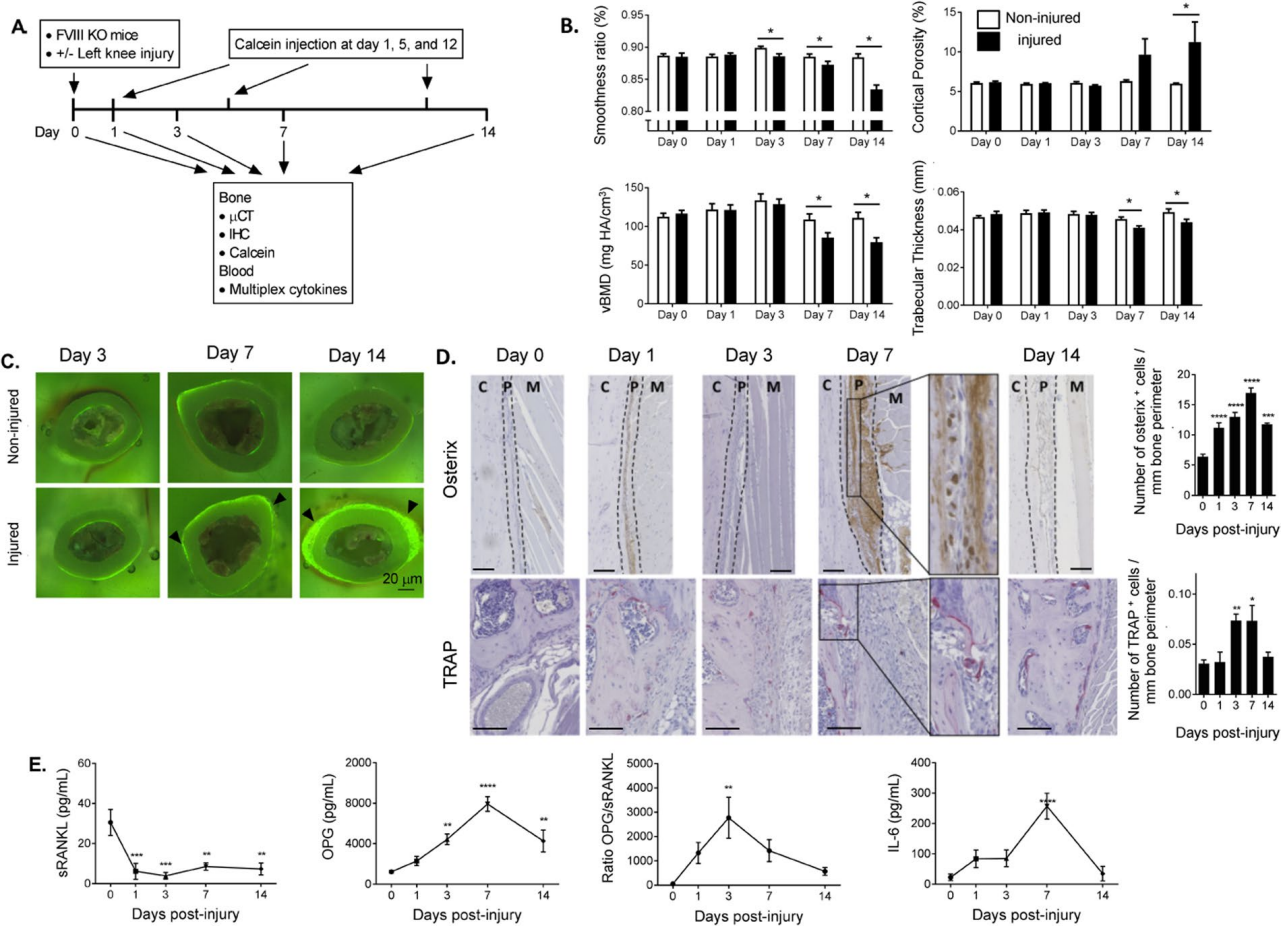
Time course study of acute lower extremity bone remodelling events following hemarthrosis in FVIII^−/−^ mice. (**A**) Experimental design of two week time course study. (**B**) The articulating bone surface showed decreased smoothness and increased cortical porosity as early as three and seven days post-injury. Integrity of trabecular bone structure decreased in terms of trabecular thickness and vBMD beginning at seven days post-injury. Both cortical and trabecular bone health continued to decline throughout the duration of the study. FWIII^−/−^ groups (WT n = 27/FVIII^−/−^ n = 34); FIX^−/−^ groups (WT n = 19/FIX^−/−^ n = 29); VWF^−/−^ groups (WT n = 18/VWF^−/−^ n = 28). (**C**) Mice were given 20 mg/kg of calcein subcutaneously as a bone formation label two days prior to euthanasia. After collection, the left femora were air-dried and embedded in molds with non-infiltrating epoxy. The resulting disks were sectioned at the mid-diaphyseal region, distal to the third trochanter. The sections were polished to a smooth surface using a combination of carbide grit paper and cloth impregnated with a diamond paste. The cross-sections were imaged at 50x magnification under UV light to visualize new surface mineralization. n = 3 per group. Calcein staining was able to detect novel heterotopic calcification at 7 and 14 days post injury, while the sensitivity of microCT allowed earlier detection. (**D**) Immunostaining of osteoblasts using osterix antibody and TRAP staining to identify osteoclasts was performed on tibia sections. Osterix^+^ cell numbers rose significantly at one day post-injury and peaked at day seven, while TRAP^+^ osteoclast numbers did not rise until day three and peaked at day seven. C: cortical bone; P: periosteum; M: skeletal muscle. Original magnification: X200. n = 5–8 per group. Scale bar: 100 μm. (**E**) Serum cytokines. sRANKL level dropped immediately post-injury, reached its lowest point at day three, and remained significantly lower than day zero values for the duration of the study. OPG levels rose significantly at three days post-injury and peaked at day seven. This resulted in an OPG/sRANKL ratio that peaked at day three post-injury. IL-6 levels peaked at day seven and returned to normal at day 14. Average ± SEM. *p < 0.05; **p < 0.01; ***p < 0.001. n = 6–8 per group.

The elevated OPG/RANKL ratio and elevated IL-6 levels are opposing forces in bone remodeling. To investigate the interface over time between these bone formation and resorption signalling pathways following injury, we performed a detailed 2 week time course study in FVIII^−/−^ mice using microCT, immunohistochemical staining, calcein incorporation, and serum cytokine assays (Fig. 6A).

MicroCT analysis detected bone changes as early as 3 days after injury, with the ratio of cortical surface smoothness worsening throughout the 2 week study and a total decline of 6% compared to the non-injured limb (Fig. 6B). MicroCT volumetric renderings indicate the surface roughening results from new, excess material forming on the exterior surface of the bone rather than degradation of existing bone^3^. *In vivo* administration of calcein confirmed that the new material is calcified (Fig. 6C). However, it was unknown if these calcifications were indicative of soft tissue mineralization or the formation of heterotopic bone by osteoblasts. Immunohistochemistry for osterix, a marker of osteoblastic lineage (osteoblast precursors and mature osteoblasts), confirmed significant increases in the number of osterix^+^ cells at the cortical surface of the bone as early as 1 day post-injury and peaking at 7 days post-injury (p < 0.0001) (Fig. 6D), and serum levels of OPG and RANKL indicated a rising OPG/sRANKL ratio from 1–3 days post injury (p = 0.0065 day 3 compared to day 0) (Fig. 6E). Additional cytokine measurements are reported in Supplemental Table 3. These data demonstrate that the earliest changes in bone health following hemarthrosis are mediated by osteoblastic formation of acute heterotopic bone surrounding the injured joint rather than merely reactive mineralization of soft tissue.

Bone resorption occurred as a second, somewhat overlapping process. Osteoclast numbers, determined by TRAP staining, increase 3–7 days post injury (p < 0.01) (Fig. 6D). At 7 days post injury, IL-6 levels spike promoting a pro-bone resorption environment (p < 0.0001 compared to day 0) (Fig. 6E)^21,22^. MicroCT measurements of vBMD and trabecular thickness decline rapidly indicating significant bone loss at day 7 and further decline at day 14 post-injury resulting in a 27% reduction in vBMD and 11% reduction in trabecular thickness compared to non-injured limb. Like the acute heterotopic bone formation, bone resorption persisted throughout the duration of the study.

## Discussion

Bone density is determined by a continuous process of coordinated bone formation by osterix^+^ osteoblastic cells and bone resorption by TRAP^+^ osteoclasts associated with a dysregulation between OPG and RANKL levels. The OPG/RANKL ratio therefore signifies the sum of bone remodeling influences at a given time, where increases in the OPG/RANKL ratio indicate a deregulated bone remodeling. Indeed, RANKL plays a pivot role in the bone resorption process by coupling RANKL producing cells (e.g. osteoblasts, osteocytes, mesenchymal stem cells, T lymphocytes) and RANK^+^ osteoclastic precursors^11,20^. RANKL is produced locally and its functional impact is tightly regulated by OPG, a decoy receptor that blocks the binding of RANKL to RANK, disrupts RANK/RANKL signalling and the osteoclastic differentiation/activation^20^. RANKL is considered as mandatory factor for osteoclastogenesis. Even if there is no clear evidence that the values of circulating OPG and RANK reflect the local production of both factors in bone, previous reports showed a correlation of OPG/RANKL ratio with the severity of bone loss^23,24^. The increase of OPG/RANKL ratio may be considered as a homeostatic response to prevent bone loss and consequently to maintain bone mass even if this system may be insufficient. Any influence that uncouples this process can result in an overall change in bone density. IL-6, a pro-inflammatory cytokine released by local inflammatory cells in response to injury, negatively regulates osteoblast differentiation^25^ and bone resorption^26,27^ through osteoblastic production of downstream effectors such as RANKL that activate osteoclasts^20^.

Low bone mineral density is an increasingly recognized complication in the severe hemophilia population. Multiple epidemiologic studies (collectively analysed in two meta-analyses) document this risk in hemophilia A adult and pediatric populations^4,5,27–30^. The independent clinical risk of hemophilia B is more difficult to determine, as most population studies have either not included hemophilia B or have not analysed hemophilia B separately from hemophilia A^28^. Studies performed by the same group of investigators and using identical methods provide an exception, separately analysing severe hemophilia A and severe hemophilia B populations, as well as a population with combined factor V and VIII deficiency, and demonstrate similar trends in bone outcomes^7,31,32^. A clinical association of low BMD with VWD has never been shown.

Separate *in vivo* investigation of FVIII^−/−^ mice by two different sets of investigators showed that complete factor VIII deficiency is associated with congenital low bone density phenotype in the absence of injury or observed haemorrhage^9,10^. The congenital bone deficits and the abnormal bone remodeling phenotype described by Liel *et al*. and Lau *et al*., respectively, were studied only in FVIII^−/−^ mice and WT mice. Therefore, no inference could be made as to whether the abnormal bone homeostasis was mechanistically attributable to a specific property of the factor VIII molecule versus defective hemostasis, inflammation, or some other more global phenomenon^3,33^. Aronovitch *et al*., in contrast, note that thrombin exerts multiple effects on osteoblasts including induction of differentiation and inhibition of apoptosis. They hypothesized that complete absence of factor VIII leads to deficient thrombin generation, resulting in ineffective thrombin-mediated signalling through protease activated receptor 1 (PAR1) and abnormal bone remodeling by PAR1-positive endosteal cells^10,22^. In support of this hypothesis, they demonstrated bone structural abnormalities were similar in mice with a complete knockout of either FVIII or of PAR1.

VWF is essential for normal platelet-to-platelet and platelet-to-matrix binding^34^. VWF also directly binds to FVIII in circulation, protecting it from proteolytic inactivation^35^. Adhesion of VWF to the subendothelial matrix at sites of vascular damage not only initiates formation of a platelet plug but brings FVIII in proximity to the site of vascular damage to promote thrombin and fibrin generation^34,36^. Recently, the FVIII.VWF complex has been shown in *in vitro* studies to interact directly with OPG to enhance its inhibition of RANKL induced osteoclastogenesis, whereas FVIII alone had no effect on RANKL mediated osteoclastogenesis^15^. We examined bone homeostasis in two strains of mice with a severe bleeding tendency due to severely deficient thrombin generation (complete knockout of either zymogen factor IX or its cofactor FVIII in the complex that activates factor X). In parallel we examined mice with a severe bleeding tendency resulting from severely deficient platelet function due to knockout of VWF. VWF^−/−^ mice have ~20% of normal circulating factor VIII (a phenotype which parallels the decreased levels of circulating FVIII in humans with Type 3 VWD, typically ~1–20%) which could exacerbate bleeding, but would be expected to support a degree of thrombin generation not present in FIX^−/−^ or FVIII^−/−^ mice^18,37–39^. We hypothesized that VWF^−/−^ mice would exhibit decreased bone density and bone resorption, particularly following injury^3,15^.

We report here for the first time that congenital deficits in bone development as well as defective bone remodeling following injury are shared by FIX^−/−^ or FVIII^−/−^ mice, as is defective bone remodeling in bones adjacent to joint hemorrhage. We found that serum OPG/RANKL ratios and low IL-6 serum levels after injury initially favour a pro-bone formation phenotype in FVIII^−/−^ and FIX^−/−^ animals. IL-6 has been associated with bone/joint health (or rather, bone/joint pathology) in hemophilia mice and and IL-6 signalling blockage by using IL-6 receptor antagonist may be used as an adjunct to replacement hemostasis and an approach to minimize hemophilic joint degeneration^40^. Accordingly, we saw increases in osteoblast number at 1 day post-injury and roughening of the cortical bone surface by 3 days post-injury. This acute heterotopic mineralization was described by Lau *et al*., at two weeks post-injury in hemophilic mice; they demonstrated the new material was porous and rough while the underlying original cortical bone maintained its density^3^. Through time course analysis we report that this new material is indeed calcified and appears in the presence of osteoblasts, implying this is acute heterotopic bone formation. Heterotopic bone formation has also been reported in hemophilia following elective joint replacement^41^. One week post-injury IL-6 levels shifted dramatically to create a pro-bone resorption environment. Osteoclast numbers elevated and trabecular thickness and vBMD dropped demonstrating significant uncoupled bone resorption particularly within the trabecular cavity. Bone formation and resorption continued in a disorganized fashion for the duration of the study. However, in the absence of observed injury we found no significant difference in BMD (Fig. 2A), BMC (Fig. 2A supplemental data), vBMD (Fig. 3B) between VWF^−/−^ and WT littermate controls (Fig. 2). Following hemarthrosis, we did find a significant decrease in cortical smoothness, lesser in magnitude but similar to FVIII^−/−^ and FIX^−/−^ mice, and no change in any other cortical or trabecular property.

Low bone mineral density was first described in severe hemophilia A in two young adults in 1994, which was the same year that the World Health Organization (WHO) developed diagnostic criteria for osteoporosis^42,43^. Despite the increasing body of epidemiologic data confirming this prevalent risk in both childhood and adult haemophilia^8,28,29^. available evidence-based and consensus guidelines for comprehensive hemophilia care do not provide guidance for bone mineral density screening or treatment for individuals with haemophilia^44,45^. Recommendations have been offered with a focus on the ageing hemophilia population and osteoporosis as a co-morbidity of aging^44–48^. Our findings of developmental bone deficits that are comparable in severely factor IX or factor VIII deficient mice not only extend previous mouse data^3,10,33^, but also demonstrate that a property intrinsic to the factor VIII protein (or the complex of FVIII with VWF) is not pivotal to the bone pathology observed in hemophilia. The findings also must be considered in the context of prior studies that demonstrate low bone mineral density and suggest primary bone pathology in children and young adults with haemophilia^49^. A limitation of the study that complicates interpretation of bone phenotype changes after injury is that the differential hemorrhage in joints is not quantified across the strains. Specifically, if the VWF^−/−^ do not bleed in this injury model, then the relative contribution of VWF to bone homeostasis after injury cannot be interpreted. The results of both the joint diameter measurements at days 1, 2, 3 and 7 after injury, and also the small but significant change in synovitis score, confirm that the VWF^−/−^ mice experience bleeding. Furthermore, the time course of the VWF^−/−^ soft tissue response to bleeding is qualitatively more like hemophilia mice than WT mice, although quantitatively less great at all time points. This is despite our results suggesting that endpoints of cortical porosity and trabecular volume/structure become abnormal in hemophilia mice with complete deficiency of FVIII or FIX (and a severe defect in amplification of thrombin generation); the same endpoints are maintained in the VWF^−/−^ mice with the severe defect of platelet associated hemostasis (combined with a mild-moderate FVIII deficiency). Our studies cannot rule out the possibility that there may be a threshold effect of bleeding, wherein mild/moderate amounts of joint hemorrhage do not significantly affect bone health until a threshold amount of bleeding is exceeded, after which the events proceed as detailed in our time course (Fig. 6). Alternatively, hemophilia mice could experience late rebleeding which prevents normal bone remodeling; the findings detailed in Fig. 6 and our time course appear to be consistent with an acute bleeding event followed by a chain of events rather than multiply recurrent bleeding insults. We believe it is likely that intact thrombin generation in the VWF^−/−^ supports bone homeostasis when compared to hemophilia, when we consider together the bone phenotype differences demonstrated in VWF^−/−^ versus hemophilia A or B in the absence of bleeding, as well as the work of other groups^10,21,22^.

In conclusion, mice with complete FIX or FVIII deficiency (but not VWF deficiency) display similarly defective congenital bone homeostasis and bone remodeling following hemarthrosis. Our results are consistent with the hypothesis that thrombin generation and signalling are important for the maintenance of normal bone phenotype. Further study of potential mechanisms of primary bone deficits in hemophilia is needed to guide appropriate screening and treatment guidelines. In addition, the effectiveness of recommended interventions for low BMD/osteoporosis in hemophilia (e.g. activity, calcium and vitamin D, anti-resorptives), if prescribed, deserve careful prospective evaluation to establish their effectiveness, in particular if implemented without prophylactic factor replacement to address hemophilia’s primary congenital deficiency.

## Methods

### Animals

All investigations were approved by the University of North Carolina-Chapel Hill Institutional Animal Care and Use Committee and were performed in accordance with relevant guidelines and regulations. FVIII^−/−^ and FIX^−/−^ mice were originally supplied by Dr. H. H. Kazazian Jr^50^. and by Dr. D.W. Stafford respectively^51^. VWF^+/-^ mice^52^ were purchased from Jackson laboratory (USA). Each knockout strain was bred in house and back-crossed 12 generations with C57Bl/6J mice. All the animals used in the experiment were male clotting factor deficient mice or male wild type (WT) littermates of the corresponding transgenic strain. At 22 weeks of age, the mice were subjected to knee joint hemorrhage induced by introducing a 30.5 needle with 5 microliters of saline into the joint space of the left knee as described previously^3,17,18^. The three strains of mice did not produced pups equally on both total numbers and numbers based on gender. The number of animals produced by colonies and used in each experiment exactly matched the real scenario at the time of experiments carried out and describe in the manuscript.

### Synovitis Scoring

At two weeks post-injury mice (n = 4–23 per group) were euthanized for histopathological examination. Mice were examined at time of necropsy for gross signs of hemorrhage. Knee joints were immersion-fixed in 10% neutral buffered formalin, trimmed, processed, sectioned, and stained with hematoxylin and eosin by routine methods. The synovium was evaluated microscopically for histopathological changes which were quantified using a 0–10 scale of murine synovitis, the Valentino scale, consisting of synovial proliferation, hemosiderin staining, villous formation, neoangiogenesis, the presence of blood, and loss of cartilage integrity as previously described^16,53^.

### DXA

Bone mineral density (BMD) and content (BMC) were assessed *in vivo* using DXA. Measurements were collected at 16, 22, and 24 weeks of age using a Lunar PIXImus Densitometer (GE Medical Systems; Madison, WI). Animals (n = 26–34 per group) were anesthetized using 2.5% isoflurane prior to and during the measurement at each time point.

### Micro-computed tomography (microCT)

Hind limbs were imaged post mortem with the knee joint intact using microCT with a voxel size of 10 microns (µCT80; Scanco Medical AG; Brüttisellen, Switzerland). The scan region spanned from the third femoral trochanter through the proximal metaphysis of the tibia. In order to quantify bone surface roughness, the proximal end of the tibia (spanning 1.5 mm) was subjected to a smoothing algorithm to remove any pits and fissures present on the surface due to heterotopic mineralization. This was accomplished by first removing any disconnected elements remaining from the joint structure. The surface of the remaining object was then dilated and subsequently eroded by 10 of voxels to produce the smoothed object. This was done using the Scanco IPL software. The surface area of the smoothed object was used in tandem with the normal, unsmoothed surface area to compute a smoothness ratio to quantify the post-injury surface mineralization. The proximal tibia was further analyzed in a region beginning immediately below the epiphyseal plate extending 1 mm distally. The trabecular bone was isolated using manually-drawn contours. Micro-architectural parameters were then measured using a visually-determined threshold of 500 mg HA/ccm of the maximum grayscale value. Cortical bone measurements were similarly obtained from the mid-diaphysis of the femur, in a volume spanning 0.5 mm axially immediately distal to the third trochanter. The threshold used was visually set at 775 mg HA/ccm of the maximum value. All measurements were obtained using the Scanco analysis software.

### Serum Markers for FVIII^−/−^, FIX^−/−^, and time course study

Serum GM-CSF, IFN-γ, IL-1β, IL-12p70, IL-13, IL-18, IL-2, IL-4, IL-5, IL-6, TNF-α, IL-10, IL-17A, IL-22, IL-23, IL-27, and IL-9 concentrations were assessed by Bio-Plex® system (Mouse Th1/2/9/17/22Treg 17plex, eBioscience, Vienna, Austria) and RANKL concentrations by using ProcartaPlex kits (Mouse RANKL Simplex, eBioscience, Vienna, Austria). OPG, DKK-2, and SOST concentrations were evaluated using Milliplex MAP kits (Mouse Bone Magnetic Bead Panel, Millipore, Molsheim, France). The procedure was performed according to the manufacturer’s protocols. Each target concentration was calculated using a 5-parameter logistic fit curve generated from the standards.

### TRAP and osterix staining

Tibias collected from euthanized mice were fixed in 4% neutral buffered paraformaldehyde and decalcified in 4.13% EDTA, 0.2% paraformaldehyde pH 7.4, at 50 °C in KOS microwave tissue processor (Milestone, Michigan, USA). Decalcified specimens were then dehydrated and embedded in paraffin. Three μm thick sections distant each from 100 µm were carried out. Tartrate-resistant acid phosphatase (TRAP) staining was performed on the tibia sections to identify osteoclasts, as previously described^54^. Immunostaining of osteoblasts was performed by using a rabbit polyclonal Osterix antibody (Abcam, Cambridge, UK), as previously described^55^. All slides were counterstained with Gill2 hematoxylin. Stained sections were automatically numerized (nanozoomer, Hamamatsu photonics) before observation with the NDP view virtual microscope (Hamamatsu). Pictures were saved using tiff format and analysed by ImageJ software (National Institutes of Health, Bethesda, Maryland, USA, http://imagej.nih.gov/ij/). A specific region of interest (ROI) of 0.5 mm beginning from the growth plate and containing most of the primary bone spongiosa was selected. Each staining was analysed to determine both relative length contact of osteoclasts to bone perimeter and osterix^+^ number (precursors of osteoblasts and mature osteoblasts) to bone perimeter (per millimeter).

### Statistical Analysis

Results were analyzed using GraphPad Prism 6.0 software (GraphPad Software, La Jolla, CA, USA). For the cytokine assays, outliers were removed using the ROUTT test, the number of samples removed is indicated in the corresponding figure legend. A one-way analysis of variance with multiple planned comparisons, or a non-parametric two-way analysis of variance (Kruskal–Wallis) was performed followed by a Sidak post-hoc test.

## Supplementary information

Supplemental Table 1

Supplemental Table 2

Supplemental Table 3

Supplemental Figure 1

Supplemental Figure 2
