# Supplementary material for: Hemophilia A and B mice, but not VWF−/−mice, display bone defects in congenital development and remodeling after injury
Source: Sci Rep. 2019 Oct 8;9:14428. doi: 10.1038/s41598-019-50787-9 (PMC6783554; doi:10.1038/s41598-019-50787-9)
Supplement: Supplementary file 2 — Supplemental Table 2 [file 41598_2019_50787_MOESM2_ESM.docx]

Hemophilia A and B mice, but not VWF^-/-^mice, display bone defects

in congenital development and remodeling after injury

Sarah Taves, Junjiang Sun, Eric W. Livingston, Xin Chen, Jerome Amiaud, Regis Brion, William B. Hannah, Ted A. Bateman, Dominique Heymann, Paul E. Monahan

**Supplemental Table 2:** Serum cytokines levels in FIX mice two weeks post-injury.

| **Cytokines** | **Statistical Significance** | | | |
| --- | --- | --- | --- | --- |
|  | **Naive WT vs KO** | **Injured WT vs KO** | **WT Naive vs Injured** | **KO Naive vs Injured** |
| IL-2 | NS | NS | NS | NS |
| IL-5 | NS | NS | NS | NS |
| IL-6 | NS | *** | NS | NS |
| IL-10 | NS | NS | NS | NS |
| IL-18 | NS | NS | NS | NS |
| IL-22 | NS | NS | NS | NS |
| IL-27 | NS | NS | NS | NS |
| DKK-1 | NS | NS | NS | NS |
| TNFα | NS | NS | NS | NS |
| sRANKL | NS | NS | NS | * |
| OPG | NS | *** | NS | ** |
| osteocalcin | NS | *** | NS | ** |

Comparisons are indicated by column titles. * *P* < 0.05, ** *P* < 0.01, *** *P* < 0.001. *NS* indicates non-significant.
