# Supplementary material for: Hemophilia A and B mice, but not VWF−/−mice, display bone defects in congenital development and remodeling after injury
Source: Sci Rep. 2019 Oct 8;9:14428. doi: 10.1038/s41598-019-50787-9 (PMC6783554; doi:10.1038/s41598-019-50787-9)
Supplement: Supplementary file 3 — Supplemental Table 3 [file 41598_2019_50787_MOESM3_ESM.docx]

Hemophilia A and B mice, but not VWF^-/-^mice, display bone defects

in congenital development and remodeling after injury

Sarah Taves, Junjiang Sun, Eric W. Livingston, Xin Chen, Jerome Amiaud, Regis Brion, William B. Hannah, Ted A. Bateman, Dominique Heymann, Paul E. Monahan

**Supplemental Table 3:** Serum cytokines levels in FVIII^-/-^ time course following injury.

| **Cytokines** | **Statistical Significance compared to Day 0** | | | |
| --- | --- | --- | --- | --- |
|  | **Day 1** | **Day 3** | **Day 7** | **Day 14#** |
| IL-2 | Day | NS | NS | NS |
| IL-6 | NS | NS | **** | NS |
| IL-12p70 | NS | NS | * | NS |
| IL-13 | NS | NS | NS | NS |
| IL-18 | NS | NS | NS | NS |
| IL-22 | NS | NS | NS | NS |
| IL-27 | NS | NS | NS | * |
| TNFα | NS | NS | * | NS |
| IFN-γ | NS | NS | NS | NS |
| DKK-1 | NS | NS | NS | NS |
| SOST | NS | * | ** | *** |
| sRANKL | *** | *** | ** | ** |
| OPG | NS | ** | **** | ** |

Each time point is compared to day 0 values. * *P* < 0.05, ** *P* < 0.01, *** *P* < 0.001, **** *P* < 0.0001. *NS* indicates non-significant. #: one outlier removed.
