# Supplementary material for: Hemophilia A and B mice, but not VWF−/−mice, display bone defects in congenital development and remodeling after injury
Source: Sci Rep. 2019 Oct 8;9:14428. doi: 10.1038/s41598-019-50787-9 (PMC6783554; doi:10.1038/s41598-019-50787-9)
Supplement: Supplementary file 4 — Supplemental Figure 1 [file 41598_2019_50787_MOESM4_ESM.pdf]

Hemophilia A and B mice, but not VWF$^{-/-}$mice, display bone defects in congenital development and remodeling after injury

Sarah Taves, Junjiang Sun, Eric W. Livingston, Xin Chen, Jerome Amiaud, Regis Brion, William B. Hannah, Ted A. Bateman, Dominique Heymann, Paul E. Monahan

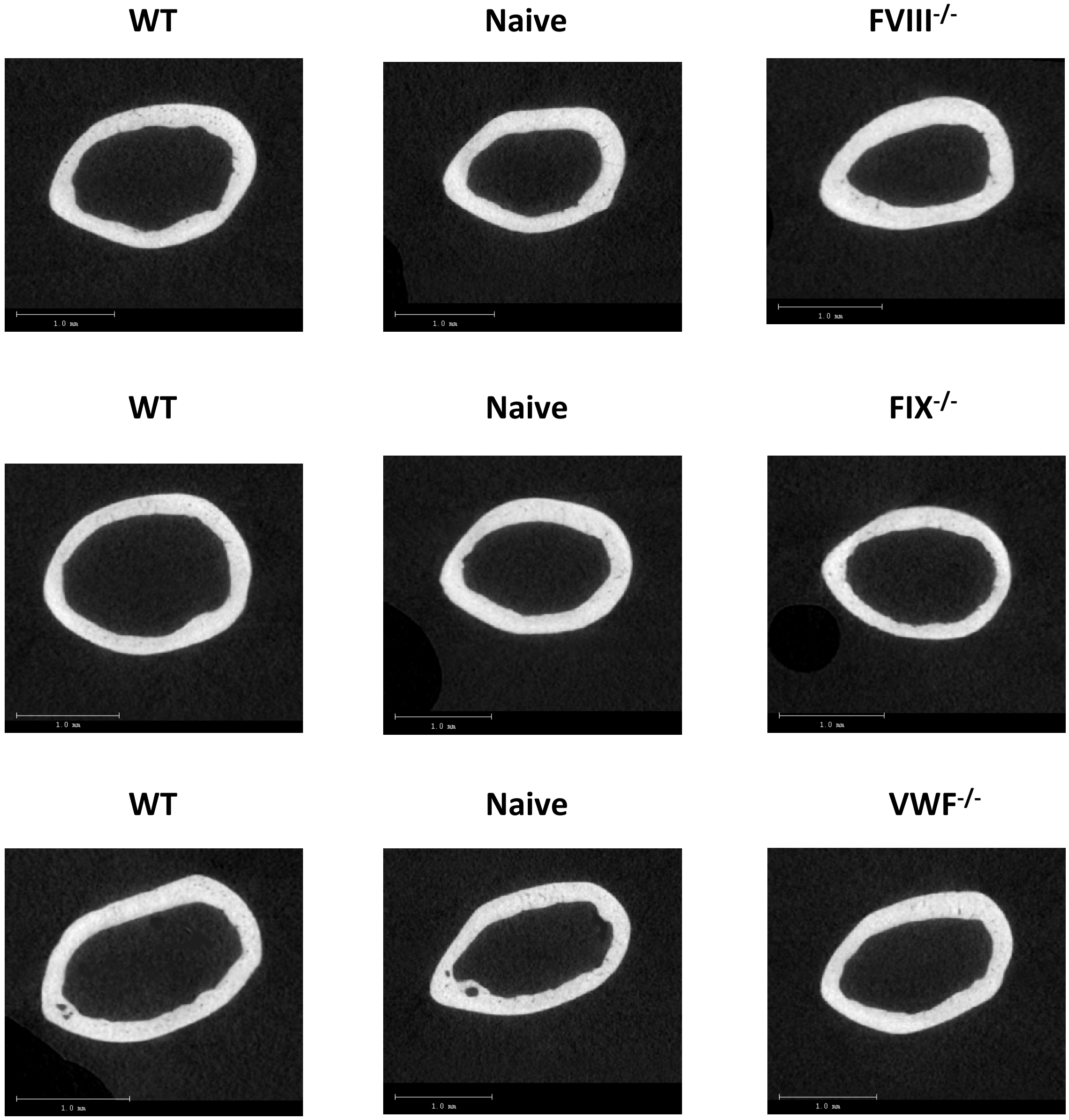

Supplemental Figure 1. Images of single slices showing the pattern of mid-diaphysis cortical porosity analyzed by micro-computed tomography.
