# Supplementary material for: Hemophilia A and B mice, but not VWF−/−mice, display bone defects in congenital development and remodeling after injury
Source: Sci Rep. 2019 Oct 8;9:14428. doi: 10.1038/s41598-019-50787-9 (PMC6783554; doi:10.1038/s41598-019-50787-9)
Supplement: Supplementary file 5 — Supplemental Figure 2 [file 41598_2019_50787_MOESM5_ESM.pdf]

Hemophilia A and B mice, but not VWF$^{-/-}$mice, display bone defects in congenital development and remodeling after injury

Sarah Taves, Junjiang Sun, Eric W. Livingston, Xin Chen, Jerome Amiaud, Regis Brion, William B. Hannah, Ted A. Bateman, Dominique Heymann, Paul E. Monahan

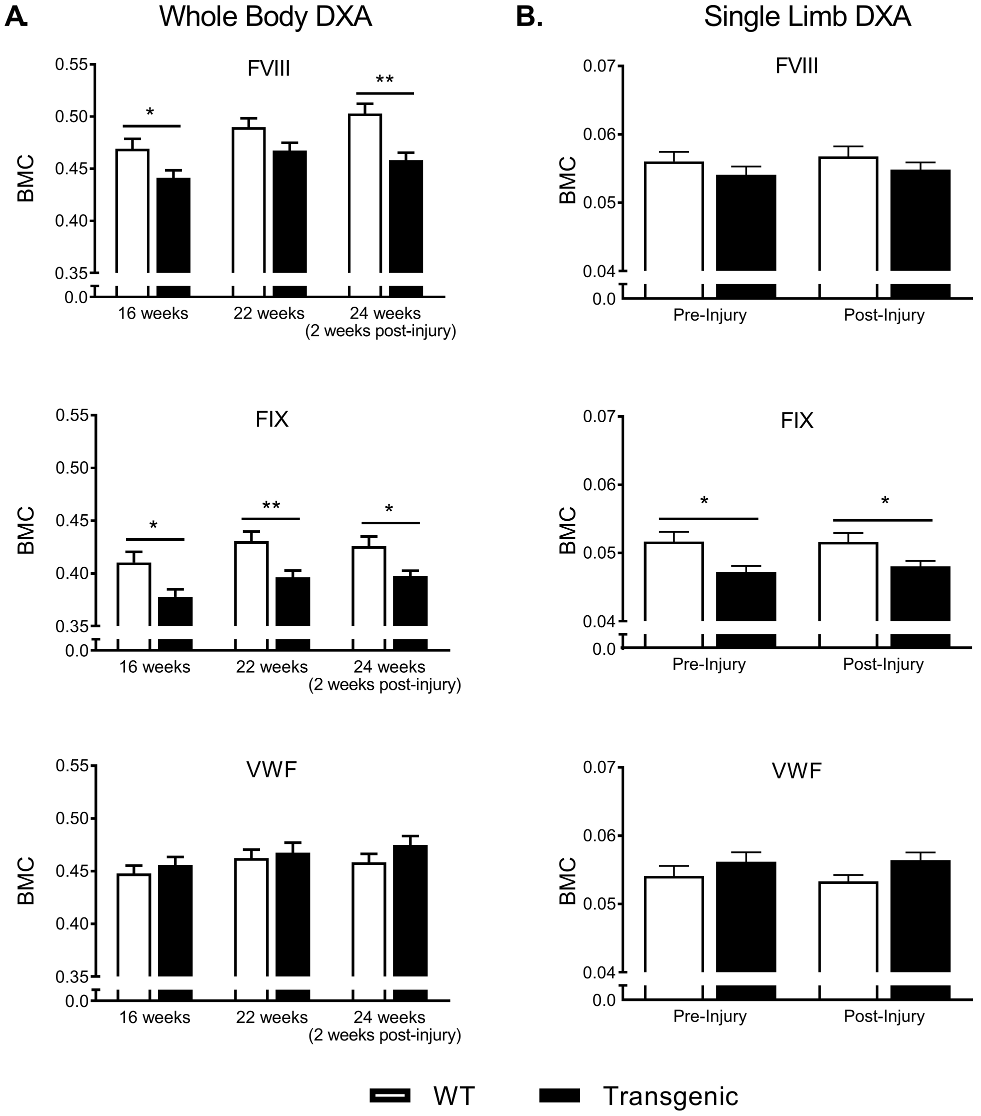

Supplemental Figure 2. FVIII$^{-/-}$ and FIX$^{-/-}$, but not VWF$^{-/-}$ mice, have significantly reduced whole body bone mineral concentration (BMC) compared to WT littermates, even prior to injury. (A) Whole body DXA analysis was performed at 16, 22, and 24 weeks of age. Between 16 and 22 weeks of age all mice show increases in BMC likely corresponding to normal growth. In comparison to WT littermate controls, FVIII$^{-/-}$ and FIX$^{-/-}$ mice showed significantly decreased BMC at 16 weeks with some recovery by 22 weeks of age in FVIII$^{-/-}$, but not FIX$^{-/-}$, mice. Two weeks following joint-injury, 24 weeks of age, whole body BMC dropped significantly in FVIII$^{-/-}$ and FIX$^{-/-}$ mice. Unlike FVIII$^{-/-}$ and FIX$^{-/-}$ mice, VWF$^{-/-}$ mice showed no difference in BMC compared to their WT littermates at any time point.

(B) DXA analysis specifically of the hind limb revealed no significant changes in BMC following injury to the left hind limb for any genotype, including the corresponding wild-type littermates. Similar to the whole body DXA data, within the FIX$^{-/-}$ cohort, KO mice showed significantly lower pre-injury BMC relative to WT littermates -9% ($p < 0.01$), which was maintained post-injury -7% ($p < 0.05$). The FVIII$^{-/-}$ group compared to WT littermates showed a similar trend, -3% pre-injury and -4% post-injury, but did not reach statistical significance. The VWF group showed no differences between knockout and WT littermate controls at any time point.

Average ± SEM. * $P < 0.05$, Average ± SEM. ** $P < 0.01$.
